# DIABRISK-SL trial: further consideration of age and impact of imputations

**DOI:** 10.1186/s12916-019-1361-2

**Published:** 2019-06-28

**Authors:** Efstathia Gkioni, Ketevan Glonti, Susanna Dodd, Carrol Gamble

**Affiliations:** 1 Department of Biostatistics, University of Liverpool, Liverpool, UK; 2 Faculty of Humanities and Social Sciences, University of Split, Split, Croatia; 3 INSERM, U1153 Epidemiology and Biostatistics Sorbonne Paris Cité Research Center (CRESS), Methods of Therapeutic Evaluation of Chronic Diseases Team (METHODS), F-75014 Paris, France; 4 Paris Descartes University, Sorbonne Paris Cité, Paris, France

**Keywords:** Randomised controlled trial, Lifestyle modification programme, Type 2 diabetes mellitus

## Abstract

Type 2 diabetes mellitus (T2DM) is a major cause of morbidity and mortality worldwide. Early interventions may help to delay or prevent onset of cardiometabolic endpoints of clinical importance to T2DM patients.

Wijesuriya et al. (*BMC Med* 15:146, 2017) published results of a randomised controlled trial in Sri Lanka testing the effect of two lifestyle modification programmes of varying intensity in participants aged 5–40 years with risk factors for T2DM. The intervention measured the impact of the two programmes on the primary composite endpoint consisting of various predictors of cardiometabolic disease. The authors concluded that the more intensive programme significantly reduced the incidence of predictors of cardiometabolic disease. Further, they delivered a large-scale intervention with restricted resources with widespread acceptance as demonstrated by the high uptake rate. However, we believe that further analysis is required to fully understand the potential for benefit, particularly in relation to age, retention and missing data.

## Introduction

There is an increasing incidence of type 2 diabetes mellitus (T2DM) in young urban South-Asians. In a large scale randomised controlled trial delivered in Sri Lanka, Wijesuriya et al. compared a trimonthly lifestyle modification programme with a less-intensive 12-monthly control programme to determine impact predictors of cardiometabolic disease in participants aged 5–40 years with risk factors for T2DM [1].

The study results were presented in two different age groups, participants aged above and below 18 years of age. We outline here a detailed explanation of why we believe that the authors, based on the sample size the research team achieved, should have provided a more detailed analysis of the different age groups. Given the rising levels of childhood obesity it is of utmost importance to understand whether resources should be focused to the different age groups.

Another important consideration given the different intensity of the interventions is the retention of participants in the clinical study and how the authors handled the missing data, so that the results are not compromised [2]. In the paragraph below about retention and missing data, recommendations have been provided about reporting the amount of missing data and the approaches that could have been used as more appropriate based on the underlying assumptions of missingness and whether they are realistic and scientifically justified.

## Age differentiation

The intervention implemented by Wijesuriya et al. [1] is generally well described; however, given the wide age range of participants included in the study (6–40 years), further details are required regarding the nature of the intervention delivered to children. It is unclear whether the study provided a nuanced intervention for those aged under 18 years considering different approaches for the various age brackets and their respective developmental stages [3]. Age-specific subgroups within the paediatric population may show differential responses to the same intervention due to their inherent physiological and educational differences; therefore, adequate power to avoid type II errors in age-specific subgroup analyses is key [4]. In their previous paper assessing the prevalence of cardiometabolic risk factors in a study population screened for randomised controlled trial participation, the authors provided demographic and anthropometric characteristics according to different age groups [5]; it is unclear why this information was not provided for the recruited study participants.

Furthermore, given that children within the lower age ranges do not have independence over their food choices and activity options, engagement of their primary carers is necessary [6]. Therefore, it would have been meaningful to provide more details on whether carers had been involved in the intervention and how the authors handled situations wherein carers and children reported differently.

Further exploration of age could also provide important information regarding the age-specific effects of the interventions on the outcomes assessed. Nevertheless, consideration of age within the statistical analysis is limited to its categorisation above or below the age of 18 years. Given the rising levels of childhood obesity and its long-term consequences [7], it is important to understand whether the intervention is equally effective across all age groups or whether resources should be targeted to particular age groups.

## Retention and missing data

In their study protocol [8], the specified follow-up period is 5 years in order to detect a 25% reduction in the relative risk between the participants in the trimonthly lifestyle modification programme and participants in the less-intensive 12-monthly control programme. However, in their final published article, Wijesuriya et al. [1] report a median follow-up of 3 years, with a range of 1 to 4 years. It would be of interest to know whether the reduction in the follow-up period was influenced by retention of study participants. More details on the group-specific retention rates would also be useful because a differential retention between groups may indicate non-adherence and biased results [9]. Given the different intensity of the interventions being compared in the study, there may be treatment group-specific differences between participant engagement and consequent retention.

Furthermore, the authors used the last observation carried forward (LOCF) method to handle missing data for participants with missing measurements. This method substitutes a single reasonable value for a missing observation assuming no change since the last observed value prior to dropout [10]. This method of imputation relies on the assumption that the probability of missing data occurs completely at random and that the probability of dropout is not related to variables such as disease severity, group assignment or intervention side effects [11]. However, the assumptions of stability and randomness may not be realistic for the study by Wijesuriya et al. [1] as the reasons causing the missing data are not known.

Imputation of a single value for the missing data is not recommended since the underlying assumptions often seem to be unrealistic and are scientifically unjustified [12]. In an anti-obesity drug trial, Jorgensen et al. [13] used different imputation methods for the missing values, including the baseline carried forward approach, where the missing weight measurements were substituted with the baseline weight, the LOCF, and the multiple imputation (MI) method, where the missing data are replaced by imputed values sampled from the predictive distribution based on the observed data. While the MI and LOCF methods in Jorgensen et al. [13] resulted in similar between-group differences for the treatment and placebo groups, this is probably because the LOCF introduces the same bias for both treatment groups. However, analysis using LOCF assumes the imputed value is known, thereby overestimating precision.

MI models impute data several times in order to allow estimation of the full uncertainty of the missing data. This method therefore incorporates not only the variability of the outcome but also the uncertainty about the missing observations. MI uses the available information to make better allowances for patients with missing data. Since the mechanism behind the missingness is unknown, and it is possible that the missing data are not missing at random [14], the MI approach [15] could provide more reliable results in comparison with the LOCF approach used by Wijesuriya et al. [1]. Bias introduced by the MI analyses could be reduced if the variables predictive of missing values are included in the imputation model.

Furthermore, in Wijesuriya et al. [1], it is unclear how many participant measurements were observed and how many were imputed. It is important for the readers to know the extent of imputation required and whether the analysis accounted for differential retention [16]. Thus, the robustness of the conclusions reached and any differences in retention rates between trial arms could be investigated in order to aid interpretation of the findings and support future trial designs.

Nevertheless, deficiencies in the reporting of missing data seem to be commonplace. Only half of the articles in a review by Rezvan et al. [17] reported both the proportion of missing data and complete cases for the variables of interest. Sterne et al. [18] also identified a lack of reporting of the MI approach, with only seven out of 59 articles reporting results from both imputed and complete case analyses. Thus, guidelines have been suggested to improve reporting of missing data analysis methods.

Despite the fact that there is no universal method for handling incomplete data in a clinical trial, there are six principles that should be considered, including the reasons causing the missingness, the primary set of assumptions about the missing data mechanism and clarification of whether the values that are missing are meaningful for analysis [12]. Although it is not possible to determine whether data are missing at random or missing not at random, sensitivity analyses addressing biases caused by data that are missing not at random are recommended to assess the robustness of findings.

## Conclusion

The DIABRISK-SL is a large, low-cost educational intervention. Therefore, it is important to take advantage of the sample size and evaluate the available information for different age groups. The analysis of participants under 18 years of age without differentiation into smaller age categories could be considered a missed opportunity to help those of an early age to establish a healthy lifestyle and prevent the incidence of type 2 diabetes mellitus. Provision of additional information regarding attrition and missing data would allow greater reassurance regarding the robustness of the results and conclusions and inform future study designs.

## Data Availability

Not applicable.

## Abbreviations

LOCF: Last observation carried forward
MI: Multiple imputation
